# A Model to Investigate Single-Strand DNA Responses in G1 Human Cells via a Telomere-Targeted, Nuclease-Deficient CRISPR-Cas9 System

**DOI:** 10.1371/journal.pone.0169126

**Published:** 2017-01-03

**Authors:** Remco P. Crefcoeur, Omar Zgheib, Thanos D. Halazonetis

**Affiliations:** Department of Molecular Biology, University of Geneva, Geneva, Switzerland; The University of Hong Kong, HONG KONG

## Abstract

DNA replication stress has the potential to compromise genomic stability and, therefore, cells have developed elaborate mechanisms to detect and resolve problems that may arise during DNA replication. The presence of single-stranded DNA (ssDNA) is often associated with DNA replication stress and serves as a signal for both checkpoint and repair responses. In this study, we exploited a CRISPR-Cas9 system to induce regions of ssDNA in the genome. Specifically, single-guide RNAs bearing sequence complementarity to human telomeric repeats, were used to target nuclease-deficient Cas9 (dCas9) to telomeres. Such targeting was associated with the formation of DNA-RNA hybrids, leaving one telomeric DNA strand single-stranded. This ssDNA then recruited DNA repair and checkpoint proteins, such as RPA, ATRIP, BLM and Rad51, at the telomeres. Interestingly, targeting of all these proteins to telomeric ssDNA was observed even in cells that were in the G1 phase of the cell cycle. Therefore, this system has the potential to serve as a platform for further investigation of DNA replication stress responses at specific loci in the human genome and in all phases of the cell cycle.

## Introduction

It is widely recognized that DNA replication stress, a hallmark of cancer, leads to genomic instability [1–5]. DNA replication stress, in the form of stalled or collapsed replication forks, can induce regions of single-strand DNA (ssDNA), which then serve as a platform for recruitment of checkpoint and repair proteins, such as RPA, ATR and ATRIP. Experimentally, regions of ssDNA can be induced in the genomc of cells by blocking DNA replication. However, to understand the response of the cell to ssDNA, it would be useful to have a method that induces ssDNA in the absence of DNA replication. In addition, inducing ssDNA at specific loci in the genome would also be desirable.

It is now possible to target specific regions of the genome by employing the clustered regularly interspaced short palindromic repeats-Cas9 (CRISPR-Cas9) system. The wild-type Cas9 protein induces DNA double-strand breaks at the targeted loci and is used for gene editing. However, one can also visualize, rather than edit, specific genomic loci in live or fixed cells by fusing a nuclease-deficient Cas9 protein (dCas9) to a fluorescent protein [6–9]. Visualization works particularly well within repetitive regions of the genome, such as telomeres, because then multiple fluorescently-tagged dCas9 molecules can be recruited to the genomic locus of interest using only one single-guide RNA (sgRNA). Targeting of specific genomic loci by dCas9 is associated with melting of the double-stranded DNA to allow formation of targeted RNA-DNA duplexes.

Here, we employed a nuclease-deficient CRISPR-Cas9 (CRISPR-dCas9) system to generate regions of ssDNA at telomeres of living cells. Our rationale was that dCas9 in the presence of sgRNA with sequence complementarity to telomeric repeats could lead to the melting of the two DNA strands, one of which would remain in a single-stranded form. Our findings suggest that the CRISPR-dCas9 system may be used to induce regions of ssDNA in the genome and, therefore, represents a promising platform for the study of specific aspects of the cellular response to DNA replication stress in a locus-specific manner.

## Results

### Specific localization to telomeres with sgRNA

**Fig 1:**
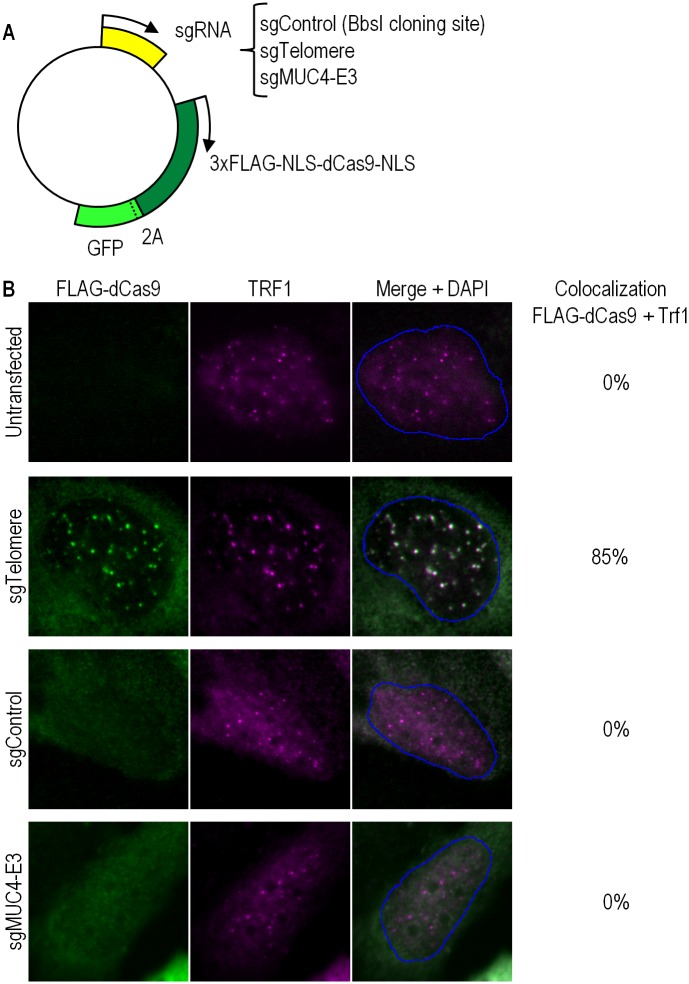
Specific recruitment to telomeres of sgTelomere RNA, colocalizing with TRF1. (a) Schematic representation of the various constructs used in this study: 3XFLAG-tagged dCas9 and a separately translated GFP marker, containing sgRNA lacking a cognate sequence in the human genome (sgControl), sgRNA corresponding to the mucin gene loci (sgMUC4-E3), or telomere sgRNA (sgTelomere). (b) An untransfected U2OS cell (upper panel) showing no detectable FLAG signal by immunofluorescence; sgTelomere transfected cell (second panel) showing nuclear FLAG foci colocalizing with TRF1; sgControl transfected cell (third panel) showing diffuse cellular FLAG signal; sgMUC4-E3 transfected cell (bottom panel) with no observable mucin gene loci.

We first sought to verify whether CRISPR-dCas9 can be targeted to telomeres as previously demonstrated [6]. To this end, we co-expressed FLAG-tagged dCas9 and a separately translated GFP marker (included for the purpose of cell sorting in subsequent cell cycle assays) using plasmid constructs, as represented in the schematic in Fig 1a. The plasmid constructs contained a control sgRNA lacking a cognate sequence in the human genome (sgControl), sgRNA corresponding to the mucin gene loci (sgMUC4-E3), or sgRNA complemetary to the telomeric TTAGGG repetitive sequence (sgTelomere) [6] (see Materials and Methods for plasmid construction). Generating extensive regions of ssDNA in the cells could be accomplished either using multiple sgRNAs complementary to consecutive sequences in the genome, or, as in our case, sgRNAs targeting the repetitive telomeric sequences. In non-transfected cells (negative control), no FLAG signal was detectable by immunofluorescence; diffuse cellular FLAG signal was detected in sgControl-FLAG-dCas9 transfected cells and, as expected, nuclear FLAG foci were seen in sgTelomere-FLAG-dCas9 transfected cells (Fig 1b). The latter foci perfectly colocalized with the telomeric repeat factor (TRF1) protein, used as a telomere marker. For unknown reasons, we were not able to detect previously described foci at the mucin gene loci in our sgMUC4-E3-FLAG-dCas9 transfected cells [6]. We therefore proceeded to further investigate only the cells in which the telomeres were targeted.

### Induction of ssDNA at human telomeres

**Fig 2:**
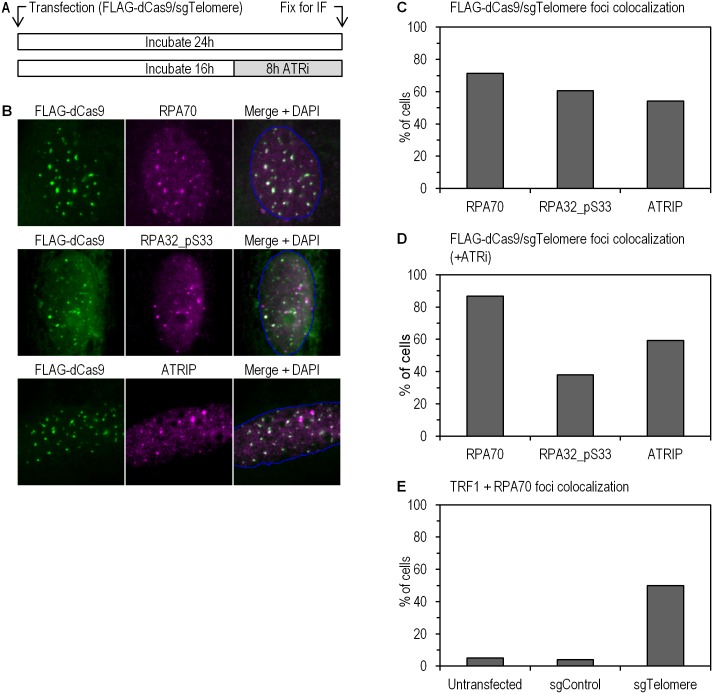
Induction of ssDNA at human telomeres. (a) Schematic description of the experimental setup, with or without 8h treatment of 10 μM ATR inhibitor. (b) Specific colocalization of sgTelomere FLAG-dCas9 with RPA70 (upper panel), RPA32 pS33 (middle panel) and ATRIP (bottom panel). (c) Quantification of cells according to foci colocalization observed in (b). RPA70, ATRIP, and RPA32 phosphorylated at S33 colocalized with sgTelomere FLAG-dCas9 in approximately 72%, 55%, and 63% of all counted cells. A total number of 120 cells per condition were counted. (d) Quantification of cells according to foci colocalization with or without treatment with 10 μM ATR inhibitor (ATRi). There was no significant effect on RPA recruitment to ssDNA (86% versus 72% without ATRi, p = 0.10), or on ATRIP recruitment to ssDNA (58% versus 55% without ATRi, p = 0.83). However, there is a significant decrease (from 63% to 38%) in the number of RPA32-pS33 foci colocalizing with FLAG-dCas9 (p<0.008). A total number of 35 cells per condition were counted. (e) Quantification of TRF1-foci-positive cells showing RPA70 colocalization. Untransfected, sgControl-, and sgTelomere-transfected cells showed, respectively, 5%, 4%, and 50% colocalization. A total number of 100 cells were counted (p<0.00001).

The first candidate to consider in the replication checkpoint machinery is naturally the replication protein A (RPA) heterotrimer, composed of RPA70, RPA32, and RPA14 subunits. Via its oligonucleotide/oligosaccharide binding (OB) motifs, RPA strongly associates with ssDNA, initiating and coordinating a cascade of molecular events critical for DNA replication integrity [10–12]. It is well established that this association between RPA and ssDNA provides a key signal in the recruitment and activation of the ataxia-telangiectasia-related (ATR) kinase, the orchestrator of cellular responses during replication stress [12–14]. We therefore examined the ATR-specific phosphorylation of RPA32 on serine 33, as well as the nuclear localization of the ATR-interacting protein (ATRIP) and RPA70 (Fig 2b). We found that, in the absence of any additional stress signal, the presence of ssDNA induced by the CRISPR-dCas9 system resulted in focal nuclear distribution of RPA70, ATRIP and the phosphorylated form of RPA32 on Ser33. All these foci colocalized with foci of sgTelomere-FLAG-dCas9. We found that RPA70, ATRIP and RPA32 phosphorylated on S33 highly colocalized with sgTelomere-FLAG-dCas9 in approximately 72%, 55%, and 63% of all counted cells (Fig 2c). These results suggest that ATM or ATR could be activated in this system. Proper ATRIP recruitment to sites of replication stress requires association between ATRIP and ATR [15, 16]. However, it has been shown that cells treated with the ATR inhibitor VE-821 still showed camptothecin-induced RPA32 foci, confirming that ATR function is not essential for DNA resection and subsequent RPA recruitment [17]. In order to validate these functional relationships in the CRISPR-dCas9 telomeric ssDNA model, we therefore repeated the above experiments in the presence of 10 μM of the ATR inhibitor VE-821 (Fig 2a and 2d). After an 8-hour treatment with the ATR inhibitor, we observed no marked effect on RPA recruitment to ssDNA, as expected, but we did observe a net decrease (from 63% to 38%) in the number of RPA32-pS33 foci colocalizing with sgTelomere-FLAG-dCas9. There was no notable difference in the fraction of highly colocalizing ATRIP foci (Fig 2d). We did not expect the latter to be significantly affected by the ATR inhibitor, since ATRIP recruitment to ssDNA has been shown to be independent of ATR [18].

In order to verify that indeed RPA foci formation corresponded to telomeric recruitment, we analyzed colocalization of RPA70 and TRF1 in untransfected, sgControl-, and sgTelomere-transfected cells. To this end, we counted the number of TRF1-foci-positive cells that showed RPA70 colocalization. Untransfected, sgControl-, and sgTelomere-transfected cells showed, respectively, 5%, 4%, and 50% colocalization between RPA70 and TRF1 (Fig 2e).

### Involvement of DNA repair pathways

**Fig 3:**
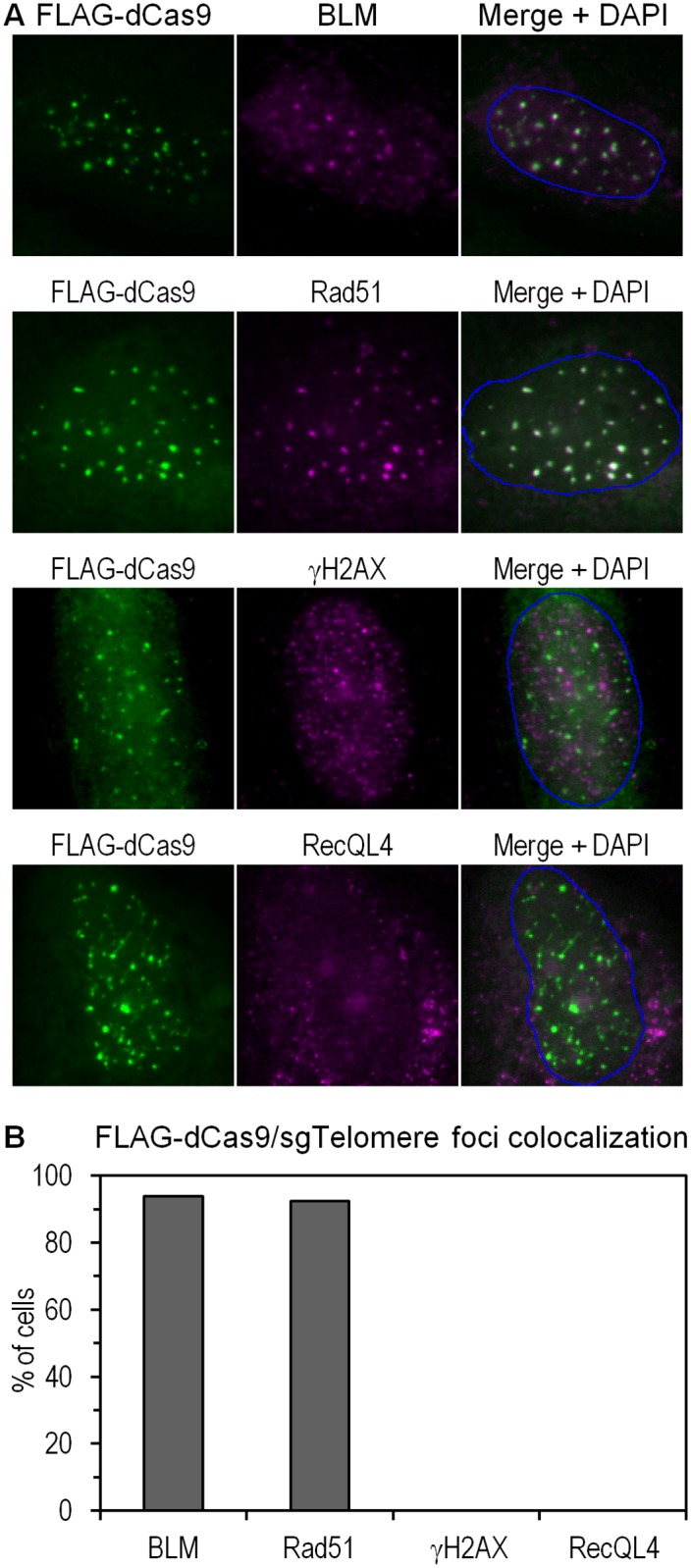
Involvement of DNA repair pathways. (a) Specific colocalization of sgTelomere FLAG-dCas9 with Bloom helicase (upper panel), Rad51 (second panel), gamma-H2AX (third panel), and RecQL4 (bottom panel). (b) Quantification of cells according to foci colocalization observed in (a). In nearly all cells, FLAG-dCas9 colocalized with Bloom helicase (95%) and Rad51 (93%). No colocalization was observed with gamma-H2AX or RecQL4. A total number of 100 cells were counted (p<0.00001).

Via its interaction with Rad51 as well as other DNA repair proteins, the Bloom helicase (BLM) has been shown to play an important role in recombinational repair [19–21]. To investigate whether our ssDNA model triggered a DNA repair response, we probed for the nuclear localization of BLM and Rad51 by immunofluorescence and observed high colocalization of both proteins with sgTelomere-FLAG-dCas9 (Fig 3a and 3b). It is worth noting that RPA itself also interacts with various proteins involved in DNA repair and checkpoint responses, including Rad51 [22] and the Bloom helicase [21]. No colocalization was observed with gamma-H2AX or RecQL4, however. This is not surprising, given the fact that gamma-H2AX is a marker of double-stranded DNA breaks [23, 24], and that RecQL4, also, has not been linked specifically to ssDNA [25, 26].

### Presence of ssDNA in G1 cells

**Fig 4:**
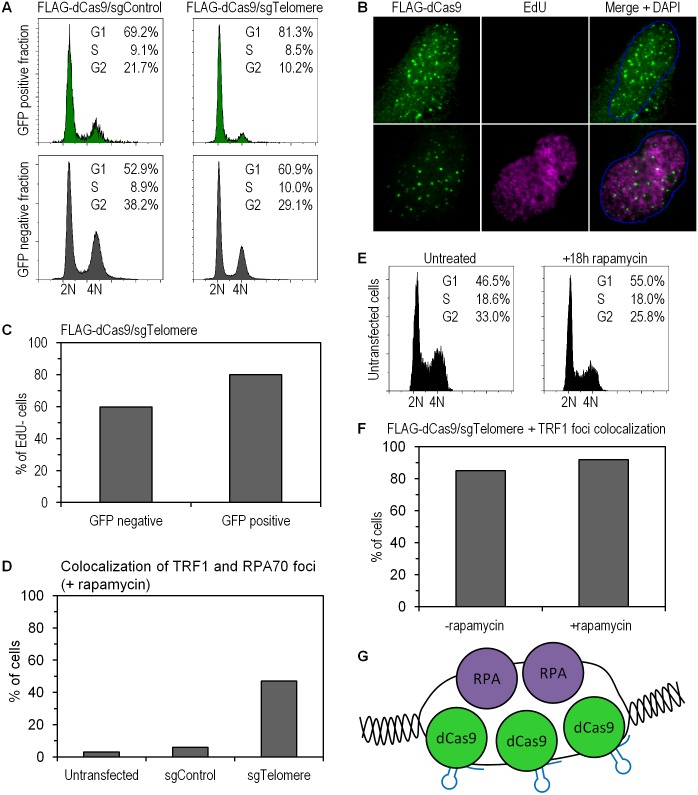
Cell cycle and EdU incorporation assays showing presence of ssDNA in G1 cells and absence of DNA replication at induced ssDNA sites, respectively. (a) A higher G1 population in sgTelomere (81.3%) versus sgControl (69.2%) was observed in transfected cells (upper panel, *GFP positive fraction*). The bottom panel describes the GFP negative fraction and serves as an internal control. (b) EdU incorporation assay showing an example of EdU positive or negative cell, co-stained with FLAG antibody. (c) Quantification of cells according to the presence or absence of EdU incorporation as determined in (b). A high proportion (approx. 80%) of FLAG-positive cells did not incorporate EdU, versus only 60% of untransfected cells. A total number of 100 cells were counted (p = 0.002). (d) Quantification of TRF1-foci-positive cells showing RPA70 colocalization after 18hr rapamycin treatment. Untransfected, sgControl-, and sgTelomere-transfected cells showed, respectively, 3%, 6%, and 47% colocalization. A total number of 100 cells were counted (p<0.00001). (e) Cell cycle profile of asynchronous U2OS cells, versus cells treated with 20 ng/mL rapamycin for 18 hours. An enrichment of the G1 fraction is observed. (f) Colocalization of TRF1 with sgTelomere FLAG-dCas9 foci in untreated cells (85% of counted cells) or cells treated with 20 ng/mL rapamycin for 18 hours (92% of counted cells). A total number of 100 cells were counted (p = 0.12). (g) Schematic depiction of a potential mechanism of sgRNA dCas9 binding to telomeric repeats in an almost uninterrupted manner, generating a string of ssDNA.

We performed Fluorescence Activated Cell Sorting (FACS) assays to investigate potential alterations in cell cycle progression in the presence of induced ssDNA. Using the separately translated GFP marker, we could selectively analyze those cells that were transfected with sgRNA-FLAG-dCas9 constructs. In asynchronous cell cultures, we observed a high fraction of cells in G1 in the populations of both the sgTelomere (81.3%) and the sgControl (69.2%) transfected cells (GFP positive fraction, Fig 4a). The fraction of GFP-negative cells in the G1 phase of the cell cycle was also high (60.9%). Similar results were obtained by monitoring incorporation of 5-ethynyl-2'-deoxyuridine (EdU), as a marker for DNA replication, in the cells expressing sgControl- and sgTelomere-FLAG-dCas9 (Fig 4b and 4c). These observations indicate that our system allows us to generate ssDNA even in non-replicating cells and, therefore the CRISPR-dCas9 system can serve as a model to study aspects of DNA replication stress, namely the presence of regions of ssDNA, even in a G1 setting.

We also treated cells with rapamycin (20ng/mL), in order to investigate whether G1 synchronization would lead to similar results observed with asynchronous U2OS cultures. RPA70 and TRF1 colocalization in untransfected, sgControl-, and sgTelomere-transfected cells was similar with and without rapamycin (Figs 4d and 2e). The effect of 18hr-rapamycin treatment, namely an enrichment of the G1 fraction as previously reported for the same conditions [27], is shown in Fig 4e. We also found out that the fraction of sgTelomere-FLAG colocalizing with TRF1 was practically unchanged with or without rapamycin treatment (Fig 4f). These results were expected given that asynchronous U2OS cells were observed to be mostly in G1, as discussed above.

## Discussion

Using the modified CRISPR-dCas9 system, an established platform for visualization of genomic sequences [6], we hereby show that it also provides a potential tool to study the DNA damage response (DDR), namely replication stress as represented by induced ssDNA. For technical reasons, our studies have solely focused on monitoring the response to telomeric ssDNA, but this of course does not preclude the potential application on any genomic sequence of interest. Specific localization of dCas9 to telomeres with sgRNA was accompanied by recruitment of the ssDNA-binding protein complex RPA to specific foci that highly colocalized with ATRIP, the BLM helicase and to a slightly lesser extent with Rad51. Immunofluorescence, cell cycle and EdU incorporation assays together provide evidence of the presence of ssDNA even in G1 phase cells.

Given that telomeres consist almost entirely of tandem TTAGGG repeats, the telomeres in our study could be bound almost uninterrupted by sgRNA-dCas9, giving rise to a along, continuous stretch of ssDNA (Fig 4g). Alternatively, we can also imagine other scenarios where genomic repeats that are more spaced apart would be targeted. This would result in multiple, distinct ssDNA sites, as opposed to a continuous stretch of ssDNA. Along the same line, yet another option would be to explore a single genomic site, instead of multiple telomeric repeats, and to determine whether there is a minimum threshold of ssDNA sites, below which no phenotype is observed.

The ability to induce the generation of ssDNA in all phases of the cell cycle, should allow one to dissect the multitude of cellular responses to DNA replication stress. Specifically, one can study the recruitment of ssDNA-binding proteins, such as RPA, in cells in G1. The recruitment of ATRIP in our system and the phosphorylation of RPA32 on Ser33 indicate that, even in G1, cells activate an ATR-dependent checkpoint response to the presence of ssDNA. Further, the recruitment of the BLM helicase and Rad51 indicate an attempt to repair this genomic lesion. We expect future studies of this system to fine tune specific locus recognition and to enable further exploration of replication stress responses, as generated by ssDNA with particular sequence specificities.

## Materials and Methods

### Plasmid construction

The plasmid containing the inactivated Cas9 gene (dCas9) flanked with N-terminal 3XFLAG and C-terminal GFP tags was constructed using pSpCas9(BB)-2A-GFP (addgene, plasmid #48138). The D10A and H840A mutations were introduced using the QuickChange Site-Directed Mutagenesis Kit (Stratagene). To enhance localization to telomeres as previously described (Chen et al., 2013), we used site-directed mutagenesis to obtain the A-U base flip in the sgRNA stem loop, and nested PCR to extend the dCas9-binding hairpin (UGCUG extension). Finally, we ligated annealed oligonucleotides into the Bbs1-restricted plasmid in order to obtain the telomeric and MUC4-E3 sgRNA sequences described in Chen et al (2013).

### Cell culture and transfection

U2OS cells (ATCC) were cultured in Dulbecco’s modified Eagle media (DMEM) (Gibco, 11960) supplemented with 10% FBS (Gibco, 10500) and 1X penicillin-streptomycin-glutamine (Gibco, 10378). For transfection, cells were split onto 18 mm coverslips inside 12-well plates (IF) (100,000 cells per well), or 10 cm dishes (FACS) (500,000 cells per dish). The next day, per 100,000 cells, 0.5 μg of each plasmid was added to a 50 μl volume of OPTI-MEM I (Gibco, 31985); FuGENE HD (Roche, 04709713001) transfection reagent was subsequently added (1.5 μl per 0.5 μg DNA). After incubation at room temperature for 25 min, this mixture was added to the cells. The ATR inhibitor VE-821 was used at 10 μM for 8 hours.

### FACS assays

GFP and propidium iodide (PI) signals were recorded from cells harvested 48 hours after transfection. PI staining was done at 5 μg/mL for one hour at room temperature. Cells were sorted on a Beckman Coulter Gallios Flow Cytometer, by the following emission wavelengths: 525 nm (GFP) and 625 nm (PI).

### EdU incorporation assays

EdU was added to the medium at a concentration of 10 μM, 2 hours before fixing the cells for immunofluorescence staining.

### Immunofluorescence

Cells were fixed 24 hours after transfection. Fixed cells were processed for immunofluorescence. The following antibodies were used: anti-FLAG (Sigma, F7425 or F1804), anti-RPA70 (Cell Signaling, 2267), anti-RPA32 pS33 (Abcam, ab87278), anti-BLM (Santa Cruz, SC-7790), anti-Rad51 (Santa Cruz SC-8349), anti-RecQL4 (Abcam, ab34800), anti-gamma H2AX (Cell Signaling, 2577), and anti-TRF1 (Santa Cruz, SC-6165). Primary antibodies were diluted 1:1000, except the in-house ATRIP antibody, which was diluted 1:20. Secondary antibodies (Alexa Fluor, Invitrogen) were diluted 1:2000.

### Imaging

Fluorescence images were acquired using a Zeiss AXIO Imager M.1 upright microscope, equipped with filter sets for DAPI, GFP and RFP (Chroma) and an X-Cite series 120 mercury vapour short arc light source (Lumen Dynamics). Images were captured with an ORCA-ER camera (Hamamatsu). An EC Plan-NEOFLUAR × 40/1.3 oil immersion objective was used for imaging of fixed cells. For data processing, the ImageJ (NIH) distribution FIJI was used. Images were enhanced by background subtraction, and linear adjustment of the image brightness and contrast.

## Supporting Information

S1 File Data values are available in the Data.xls file. (XLSX)
